# Can productivity growth measures identify best performing hospitals? Evidence from the English National Health Service

**DOI:** 10.1002/hec.3847

**Published:** 2019-01-18

**Authors:** María José Aragón Aragón, Adriana Castelli, Martin Chalkley, James Gaughan

**Affiliations:** ^1^ Centre for Health Economics University of York York UK

**Keywords:** England, growth, hospital, NHS, productivity

## Abstract

Health‐care systems around the world face limited financial resources, and England is no exception. The ability of the health‐care system in England to operate within its financial resources depends in part on continually increasing its productivity. One means of achieving this is to identify and disseminate throughout the system the most efficient processes. We examine the annual productivity growth achieved by 151 hospitals over five financial years, using the same methods developed to measure productivity of the National Health Service as a whole. We consider whether there are hospitals that consistently achieve higher than average productivity growth. These could act as examples of good practice for others to follow and provide a means of increasing system performance. We find that the productivity growth of some hospitals over the whole period exhibits better than average performance, but there is little or no evidence of consistency in the performance of these hospitals over adjacent years. Even the best performers exhibit periods of very poor performance and vice versa. We therefore conclude that accepted methods of measuring productivity growth for the health system as a whole do not appear suitable for identifying good performance at the hospital level.

## 1 INTRODUCTION

Expenditure on health care delivered by the National Health Service (NHS) is one of the largest budget items in the U.K. public sector, amounting to over £100 billion each year since 2009/2010 and reaching £120 billion in 2016/2017. Recent experience is that the demand for health care outstrips the willingness to increase taxes to finance it, giving rise to a funding challenge. Increased *productivity*—a greater flow of treatments and health care for a given expenditure on inputs—is seen as an important element in meeting that challenge. Previous research by Bojke, Castelli, Grašič, Howdon, and Street (2016) has measured productivity growth across the whole NHS system between 2012/2013 and 2013/2014 and found that productivity increased approximately by 2%. The aspiration is that the NHS should double that rate in order to meet increasing demand within the financing regime that has been agreed for it. One focus in seeking that improvement is to consider whether some producing units, that is, NHS *Trusts*, that are delivering only modest increases can be induced to emulate those that are already exceeding the requirement. The Carter Review highlights the importance of keeping track of Hospitals' productivity in order to ensure that the improvements required by 2020 are being made (Lord Carter of Coles, 2016). We examine whether the application of productivity growth measurement, as has been applied to the whole *system*, permits the identification of high and low performing producing units. Our specific focus is on provision of hospital (secondary) health services by organisational units called NHS *Trusts*, 1Trusts can provide their services at more than one site, but for the analysis, we consider all the activity of a Trust together.which we henceforth simply term *hospitals*. We address the question of whether the measurement of productivity growth scales down to individual hospitals. If applicable, such methods could be used to identify high performers and use these as examples of good practice for the whole system.

As with many forms of production, hospital health care involves multiple outputs, produced using multiple inputs. We aggregate the different inputs and outputs using methods that have been derived and utilised for measuring productivity in the English health‐care system as a whole and construct measures of the annual growth in productivity—the differential growth of output to input—of 151 hospitals for five pairs of years. Over the full study period, the hospitals show substantial differences. Some exhibit growth in productivity and others decline. There are also differences between hospitals in the growth they have each year and over time: Between‐hospital differences in year‐by‐year growth rates are smaller than the within‐hospital year‐on‐year differences. Hence, there is little if any *consistency* in the productivity performance of hospitals over time.

This work extends past research into levels of productivity in England's hospitals (Aragón Aragón, Castelli, & Gaughan, 2017; Castelli, Street, Verzulli, & Ward, 2015) by considering productivity growth at the hospital level. Measures of productivity levels are strongly affected by differences between hospitals that do not reflect performance. For example, a hospital that has inherited a large or overvalued capital stock will have lower productivity. Further, there are regional differences in the allocation of capital to hospitals within the health system we study on account of structural reorganisation. By considering productivity growth, such effects are differenced‐out along with any other hospital‐specific idiosyncrasies in valuing or accounting for inputs or outputs. Furthermore, the focus of policy is on improving performance, and this is more directly measured through growth in productivity.

There is a considerable literature that uses some form of frontier analysis (data envelopment analysis [DEA] or stochastic frontier analysis [SFA]) to measure productivity at the system or unit level (see Hollingsworth, Dawson, & Maniadakis, 1999, for a review of methods). Frontier models work under the assumption that the existing technology is not fully utilised by the production units. The main advantage of frontier models is their ability to disentangle the source of efficiency change: technological change versus technical efficiency change (Del Gatto, Di Liberto, & Petraglia, 2011). Another advantage is the capacity to compare inputs and outputs in different units such as staff number or number of beds in a hospital. However, as the productivity frontier constructed by such methods has a dimension for each input or output included, SFA and DEA approaches are most effective when the number of observed units is large relative to the number of input and output types to consider. In our case, the number of different inputs and outputs is large, and the number of observed units (hospitals) is relatively small. Alternatively, we might apply frontier methods after converting the many observed dimensions of inputs and outputs into a single dimension for each; the method to do this is discussed below. However, using a single input and output dimension would remove the very advantage of frontier methods to analyse multidimensional measures of inputs and outputs.

The index numbers approach, as other non‐frontier methods, assumes that production is always technologically efficient (Del Gatto et al., 2011). The index approach has the main advantage that it does not rely on a parametric specification of the model (as does SFA). This makes it particularly amenable when measuring the productivity of whole health‐care systems for which it is difficult to correctly specify a unique production function. The health‐care system comprises a number of smaller production units (hospitals, GP practices, etc.), all of which have their own production function. A further advantage of the index approach is the conversion of many inputs and outputs with different units into a common unit of financial value. The most used index numbers are the Laspeyres, Paasche, Fisher, and Tornqvist (Diewert, Balk, Fixler, Fox, & Nakamura, 2010).

Index numbers, such as the Laspeyres and Paasche indices, are used in National and International Accounts when measuring whole system and private/public sector productivity growth over time. Similarly to the DEA approach, in the National Accounts, productivity is defined as the ratio of outputs to inputs. Outputs are usually differentiated to reflect their differing quality, which is in turn reflected in the prices of the products. When considering the output of the public sector, (market) prices do not usually exist. It is common practice to use cost weights to aggregate different forms of activity into a single measure of output growth. This approach has permeated the measurement of productivity in the English NHS (Castelli et al., 2007; Dawson et al., 2005), and it is the approach we adopt.

In this setting, the most recent work by Bojke, Castelli, Grašič, and Street (2017) considers the growth in NHS outputs, inputs, and productivity between 1998/1999 and 2013/2014, and they find that outputs have increased more than inputs, 89% versus 82%, and therefore, productivity has increased, by 4%, over the 15‐year period. Castelli et al. (2015) and Aragón Aragón et al. (2017) adapted the methodology developed by Dawson et al. (2005) for measuring whole system productivity growth over time to the measurement of health‐care productivity of single hospitals in a cross‐sectional context. Further, they used hospitals' productivity ratios (standardised around the national average) to uncover possible drivers of productivity variations across different providers. Castelli et al. (2015) found extensive variations across hospitals, ranging between −62% and +33% below/above the national average across the years 2008/2009 and 2009/2010, which are highly correlated over time. Aragón Aragón et al. (2017) also found substantial variation in hospital‐level productivity, both in the labour productivity and total factor productivity (TFP) measures, for the financial years 2010/2011, 2011/2012, and 2012/2013. In particular, they found productivity ratios to vary between −36% and +79% for the labour productivity measure and between −50% and +33% for the TFP measure. Distinct from these contributions, we focus on *growth* and thus difference‐out any time‐invariant hospital‐level determinants of productivity, with a view to uncovering robust underlying differences in the performance of hospitals. We find that, unlike productivity levels, productivity growth is not correlated over time.

The rest of this paper is set out as follows. A description of the core methodology for measuring productivity and data used are set out in Section 2. Results are described in Section 3. Key findings and conclusions are set out in Section 4.

## 2 METHODS AND DATA

This was a retrospective analysis of previously collected, non‐identifiable information and involved no change in the management of patients. Obtaining individual consent was not feasible, and patient records are pseudonymised and de‐identified by NHS Digital prior to release for research.

TFP growth of each hospital is calculated by combining data on the array of outputs produced and inputs used. We construct an output growth index (*X*) and an input growth index (*Z*), 2Both *X* and *Z* are indices with values around one, for example, 1.02 indicates a 2% increase and 0.99 indicates a 1% decrease. Therefore, the productivity growth calculated using them will also be an index, which can be transformed into a percentage by subtracting 1 and multiplying by 100.with TFP growth, △*T* *F* *P*, calculated as the ratio of the growth of outputs produced to the growth of all inputs used in its production (Bojke, Castelli, Goudie, Street, & Ward, 2012):

(1) $$△TFP=\left[X/Z\right].$$

Using year‐on‐year productivity growth rates, we can assess productivity growth over longer periods of time for each hospital by means of a chained index:

(2) $$\prod_{t=0}^{T}△TFP=\frac{X_{\left(0,t\right)}}{Z_{\left(0,t\right)}}\times \frac{X_{\left(t,t+1\right)}}{Z_{\left(t,t+1\right)}}\times \text{⋯}\times \frac{X_{\left(T-1,T\right)}}{Z_{\left(T-1,T\right)}},$$

where each link of the chain is represented by Equation (1) for the relevant two consecutive years. From here onwards, we will use the term *link* to refer to any two adjacent years over which we calculate growth.

To estimate TFP, it is necessary to define and measure both outputs produced and inputs used for each hospital. Growth in both of these can be calculated either directly or indirectly (Eurostat, 2001; OECD, 2001). A *direct* volume measure aggregates information about the volume of each type of output (input) produced (used) using their prices as weights. An *indirect* measure usually relies on other type of information, most often expenditure data.

Following Dawson et al. (2005), 3Dawson et al. (2005) developed an NHS output index that was utilised in the Atkinson Review (Atkinson, 2005) and is also used in the U.K. National Accounts (Office for National Statistics, 2009, 2012). Unlike Dawson et al. (2005), where the NHS output index in the National productivity series is adjusted for quality of hospital inpatient activity, namely, survival rates adjusted by life years gained and waiting times, our NHS output growth index does not include a quality adjustment for the hospital inpatient component. This decision was based on conclusions reached in Aragón Aragón et al. (2017) that the above indicators would introduce further noise to the hospital Trust level productivity estimates.we construct a set of paired year‐on‐year Laspeyres output, input, and productivity indices at hospital level. Outputs consist of all health‐care goods and services delivered to NHS patients in one of what are termed care *settings*. Examples of these include outpatient services or inpatient spells. As NHS goods and services are delivered free at the point of use, the index uses unit costs as weights, instead of prices, to aggregate the different types of outputs produced. This approach is in line with recommendations set out in the National Accounting literature (Atkinson, 2005; Eurostat, 2001). Inputs into the health‐care system consist of labour, intermediate goods and services, and capital. As no comprehensive volume data on all factors of production are available, we employ a *mixed* method in determining the growth in these inputs. This combines a direct volume measure of labour input (excluding staff that are engaged on a temporary basis, termed *agency staff*) and an indirect measure, relying on expenditure data, for agency staff, intermediate goods and services, and capital. This also follows recommendations set out in the National Accounts (Eurostat, 2001; World Bank, 1993). Given that expenditure is driven by both the volume and price of inputs and our focus is on determining the change in the volume of inputs used in two adjacent years, we need to isolate changes in the volume of inputs used from changes in their prices. To this end, we construct a measure of *constant* expenditure using a price deflator for each input.

It is well understood that the availability and sources of data used in constructing output and input growth indices may change over time. We therefore adopt the “imputation” method developed by Castelli, Laudicella, Street, and Ward (2011) to account for these issues in our data.

**Figure 1:**
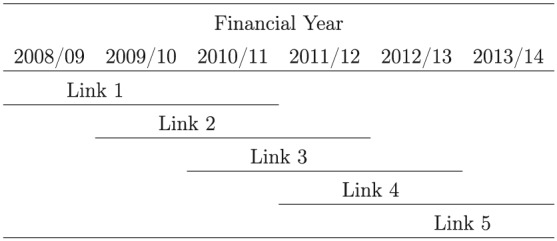
Financial years and links

This study covers six financial years from 2008/2009 to 2013/2014; thus, we present results for five pairs of consecutive years *(links)*; see Figure 1.

Two datasets are used to calculate the volume and unit costs of the services provided by each hospital: Hospital Episode Statistics and Reference Costs. We use the Hospital Episode Statistics dataset to obtain information regarding levels of inpatient activity and the Reference Costs dataset for both activity and unit costs information for all non‐inpatient settings (see Table 1 for a list of activity settings), as well as for the unit costs of hospitals' inpatient activity.

**Table 1:** Settings considered in each link

	Average share of output (%)	L1	L2	L3	L4	L5
Number of hospitals		151	151	151	151	151
Inpatient	53.03	151	151	151	151	151
Outpatient	19.77	151	151	151	151	151
A&E	4.54	138	138	138	138	138
Chemo/Radiotherapy	5.45	149	149	149	148	150
Community care	2.60	136	138	136	135	134
Community mental health	0.17	25	23	.	24	26
Diagnostic tests	2.08	133	136	136	141	142
Radiology	2.12	149	149	150	149	150
Rehabilitation	1.07	79	76	76	85	84
Renal dialysis	1.32	59	59	60	56	59
Specialist services	7.12	148	148	148	148	148
Other	0.73	138	141	140	140	142

*Note*. In link L3 (2010/2011 to 2011/2012), Community mental health was not included because this setting was affected by a complete overhaul in 2011/2012, which resulted in the incomparability of the mental health activity in the two financial years.

There are two main sources of data in terms of inputs, the Electronic Staff Record and the hospitals' financial accounts. The Electronic Staff Record provides information on NHS staff numbers, measured in terms of full‐time equivalents, for over 480 different types of NHS staff groups. 4The number of different types of NHS staff has increased over the time period considered. The figure earlier refers to the most recent financial years. The corresponding earnings data are extracted from the NHS Payroll and Human Resources system. The expenditures reported in hospitals' financial accounts are grouped into labour (where possible distinguishing NHS staff from agency staff 5Expenditure on agency staff was reported separately by non‐Foundation Trusts until 2012/2013. Since then, they report only one labour expenditure entry, covering permanent staff. Thus, we use agency expenditure data provided by the Department of Health.), intermediates, and capital. We use two deflators from the Hospital and Community Health Services pay and price data to deflate input expenditures—specifically, the Pay Cost Index to deflate agency staff expenditure and the Health Service Cost Index to deflate intermediate and capital expenditures.

A more detailed explanation of the methods and data used in the analysis is available in Aragón Aragón, Castelli, Chalkley, and Gaughan (2016).

We focus on the subset of hospitals that did not undergo any organisational change during the period of analysis, that is, those that existed in the financial year 2008/2009 and have not merged or ceased to exist by the end of the financial year 2013/2014. 6The total number of hospitals ranges between 160 and 166 during the period of analysis; 151 of them remain unchanged throughout, that is, do not undergo any mergers or dissolutions over the time period covered in this study.This approach ensures that we calculate the productivity growth measure for hospitals that are not a priori expected to show any given growth rate in a given period, whereas those that merge are expected to change their growth rates after the merger takes place.

Table 1 shows how many of the 151 hospitals considered in this study have activity in each of the settings in each link.

## 3 RESULTS

**Figure 2:**
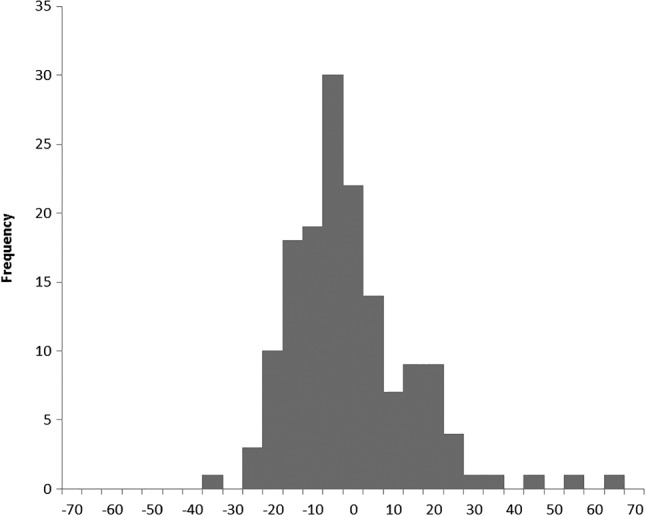
Distribution of overall growth of the productivity measure. Unchanged hospitals. Note that the bins are labelled using the upper limit of the interval they represent, for example, the bin labelled “0” corresponds to the interval [−5, 0)

Table 2 shows the productivity growth between 2008/2009 and 2013/2014 calculated using the chained index from Equation (2), and Figure 2 shows its distribution.

**Table 2:** Productivity growth rates

	2008/2009 to 2013/2014
Number of hospitals	151
Min	−31.63
25th percentile	−8.79
Median	−1.34
75th percentile	8.83
Max	67.16
Mean	1.41
Standard deviation	14.90

Note. Descriptive statistics and distribution.

From Table 2, overall measured growth in productivity over time is on average positive. Its distribution is not symmetric (mean and median do not coincide), and there are more hospitals with negative overall growth than with positive overall growth (median is negative). The mean productivity growth over the period 2008/2009 to 2013/2014 is 1.4%; this growth is similar to that estimated by Lafond, Charlesworth, and Roberts (2015), who calculated productivity for the period 2009/2010 to 2013/2014 considering only inpatient and A&E and found the productivity growth over that period to be 1.3%.

Figure 2 suggests that overall growth has a distribution that corresponds to a conventional, approximately bell‐shaped, curve. At face value, this would suggest that there are good and bad performing hospitals (the tails of the distribution), which could be used as either exemplars of good performance or investigated for poor performance. That approach however assumes that there is something enduring about the performance of hospitals, such that *overall* productivity growth over time is at least approximately similar over shorter periods. The remainder of our analysis establishes that this assumption is violated.

**Figure 3:**
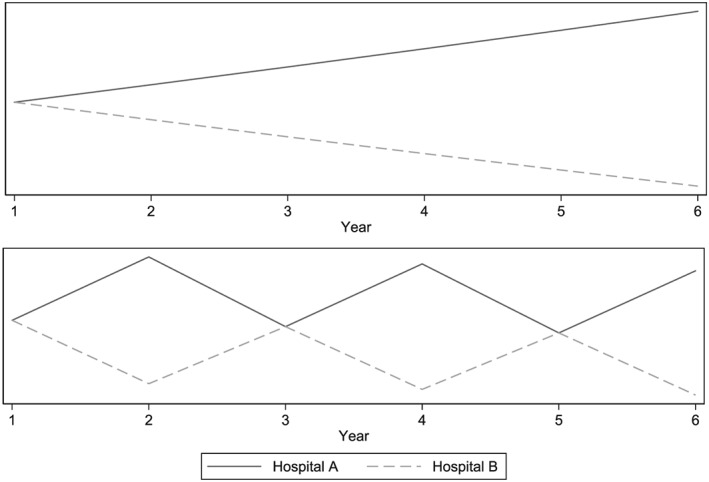
Hypothetical growth paths

Figure 3 presents two stylised patterns of productivity growth. Each panel shows two providers, both beginning with the same level of productivity. In the top panel, Hospital A has a consistent growth rate of 2% per year and Hospital B a consistent negative growth rate of 2% per year. This panel represents the assumption that growth over time is consistent and ordering in terms of growth is independent of the specific time period considered. In the bottom panel, both hospitals follow the same pattern of growth, alternating between a 10% increase and 10% reduction in productivity on consecutive years. The only difference between hospitals in this panel is that between Year 1 and Year 2, there is an increase for Hospital A and a decrease for Hospital B. This pattern gives the same ordering of growth over the study period as the top panel after 5 years (i.e., growth between Year 1 and Year 6). However, if we were to calculate growth between Year 1 and Year 5, we would find no difference between the hospitals. The bottom panel violates the assumption that ordering in terms of overall growth is independent of the study period selected.

**Figure 4:**
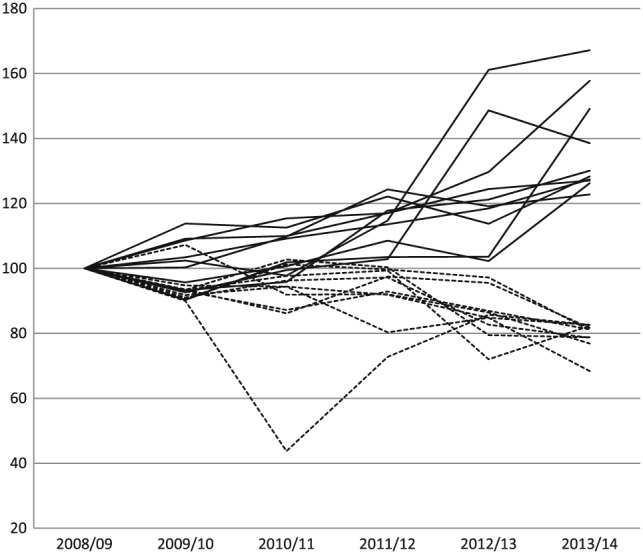
Productivity levels (2008/2009 = 100). Hospitals with the highest (solid lines) and lowest (dashed lines) overall growth

Figure 4 shows changes in productivity growth for the 10 hospitals with the highest (solid lines) and the 10 hospitals with the lowest (dashed lines) overall growth. 7We focus on the 10 hospitals at either extreme to simplify the plot.Figure 4 is constructed by fixing the initial productivity *level* of every hospital to be 100 and using the measured growth in productivity for each link to calculate their productivity in each of the following financial years. This figure indicates a high degree of variation in productivity over time for individual hospitals, rather than a smooth growth path. Therefore, the overall performance of hospitals is strongly influenced by the choice of starting/ending points of the time period considered for the evaluation. Over different intervals of time, a hospital may appear to be among the better or worse performing providers.

Figure 4 shows the great variability of productivity growth for any one hospital over time. Because good or poor performance is a relative concept, another way of describing variation in performance over time is to calculate the transition probabilities of moving from one quartile of growth performance to another. Quartiles are defined by the minimum, 25th percentile, median, 75th percentile, and maximum growth rates in each link, with Q1 being the quartile between the minimum and the 25th percentile and Q4 the quartile between the 75th percentile and the maximum. Table 3 shows these probabilities as percentages. Rows reflect the initial (link *t*) quartile, and the columns reflect the final (link *t* + 1) quartile. If growth in one link was independent from growth in the previous link, we would expect a uniform random distribution of hospital growth level, with 25% in each quartile.

**Table 3:** Transition probabilities

		Quartile in link *t* + 1			
		Q1	Q2	Q3	Q4
Quartile in link *t*	Lowest growth ‐ Q1	21.05	14.47	25.00	39.48
	Q2	24.32	29.05	26.35	20.28
	Q3	23.03	25.66	26.32	24.99
	Highest growth ‐ Q4	32.24	28.95	23.03	15.78

The first row of Table 3 shows that 21% of hospitals that had the lowest values of growth in their productivity measure (Q1) in one link are also among those with the lowest growth (Q1) in the following link; 14% of hospitals have a growth rate lower than the median growth (Q2) in the following link; 25% of hospitals have a growth rate higher than the median (Q3) in the following link; and 39% of hospitals are among those with the highest growth (Q4) in the following link. This strongly reaffirms the lack of consistency in respect of a hospital's productivity growth.

We next consider whether the probability of remaining in the same growth quartile depends on the hospital's initial position. In particular, we are interested in finding out whether hospitals with productivity growth measures falling into either Q1 or Q4 move more frequently to a central quartile (Q2 or Q3) in a subsequent period rather than remaining in an extreme quartile (Q1 or Q4). This type of pattern is generally referred to in the literature as regression to the mean. Our results show that the most likely quartile following an extreme (Q1 or Q4) is the opposite extreme; we find that the probability of moving from Q1 to Q4 is 39% and that the probability of moving from Q4 to Q1 is 32%.

Overall, these results give no indication of persistence in productivity growth. Similar results hold when restricting the sample of hospitals to those that do not experience extreme (more than three standard deviations away form the mean) growth in either outputs or inputs in any link or when considering labour productivity (Aragón Aragón et al., 2016).

## 4 DISCUSSION

Productivity—the effectiveness of converting inputs into outputs—is a fundamental and recurring concern for health‐care systems and is a particular focus of attention for a publicly funded resource‐constrained system such as the NHS in England. Extensive previous research has established the means of measuring productivity and documenting its growth at the system level. This methodology underpins important policy decisions in respect of future resourcing, with the health‐care system being given productivity targets as a condition of sustained increased funding. More recently, attention has turned to how those targets can be met. One approach is to identify the units that are performing well, with a view to using those as examples for others to emulate. In this paper, our focus is on a very substantial component of the health‐care system—hospitals—and we consider whether the tools and methods of whole system productivity measurement can be utilised to establish productivity at the unit (hospital) level.

Our approach is designed to give the methods the best chance of success. We adopt the most extensive data and combine it using the methods and experience developed over more than 10 years of productivity analysis. Our focus on measuring growth of productivity removes one major limitation arising from hospital‐level analysis that specific and time‐invariant factors might inflate or deflate measures of productivity for particular hospitals. This may be due, for example, to idiosyncrasies in accounting methods or having inherited inflated endowments of inputs (especially relevant in the case of capital). Furthermore, we use a chained index method to insulate our calculations from homogenous system‐wide changes.

Notwithstanding these attempts to ensure the best and most consistent measures of productivity performance at the hospital level, our findings indicate very little consistency in measured performance. In terms of productivity growth, good performance is more often followed by poor performance than further good performance and vice versa. This casts considerable doubt on the utility of productivity growth measurement at the level of individual hospitals. Without confidence in a measure of performance and persistence in the ordering of that measure, it is not plausible to identify “good” performers or “bad” performers. Although it is clearly tempting to think that the process of adducing evidence in respect of productivity growth for the whole health system might provide evidence of performance of its constituent components, we have established that this is not the case for hospitals and our findings provide a stimulus for further research into the question of how to measure performance at a more micro level. Our findings are also relevant to the question of what organisational unit should be subject to investigation, because it is possible that although productivity growth does not produce consistent performance measures at the level of the hospital, it might be relevant at other less aggregate levels than the national health system, for example, regional health systems or specific clinical departments.

The failure to identify consistent performance across hospitals does not diminish the value of system productivity measurement. The health‐care system is composed of a large number of producing units, and if the measurement of performance at the unit level is subject to high variance and serially uncorrelated shocks, the aggregate measure will still be a true reflection of system performance. It is worth stating that our study has not found a particular cause of volatility in productivity measurement at the hospital level. Decomposing outputs and inputs does not reveal one particularly noisy data element. Instead, we find substantial noise at the level of individual hospitals in terms of both inputs and outputs. One potential mechanism behind this observation is heterogeneous effects of national policies. We exclude hospitals that underwent mergers or dissolutions in the study period, but these are not the only institutional changes that can occur, and the effect of these need not be homogenous. For example, the removal of community services from Primary Care Trusts came into effect during our study period. Responses to this policy varied, with some new Community Trusts being created but in other cases hospitals taking over more community care. Such differences can be expected to lead to differences in inputs and outputs at the hospital level, as such changes represent changes in attribution to hospitals. However, at the system level, reattribution per se does not affect productivity across the whole. A second potential mechanism for noise is changes to the *attribution* of inputs and outputs. Suppose that, for example, aggregate inputs are fixed but are allocated differently to individual hospitals in successive time periods, then we would expect to observe potentially very large changes in measured individual hospital productivity but not change in overall productivity. This is broadly consistent with what we have measured.

Further research would be needed to identify the sources of volatility and examine whether there are alternative measures of productivity with sufficient consistency to guide interventions to improve performance at the hospital level. One potential line of inquiry is to investigate for which hospitals available measures of productivity growth are more reliable. For example, in Aragón Aragón et al. (2017), the relationship between productivity levels and hospital size, as well as other hospital characteristics, has been highlighted as relevant to overall performance. Such differences might also impact on measure reliability, making existing measures more or less appropriate for different subsets of hospitals.
